# Natural 5-Aminolevulinic Acid: Sources, Biosynthesis, Detection and Applications

**DOI:** 10.3389/fbioe.2022.841443

**Published:** 2022-02-25

**Authors:** Meiru Jiang, Kunqiang Hong, Yufeng Mao, Hongwu Ma, Tao Chen, Zhiwen Wang

**Affiliations:** ^1^ Frontier Science Center for Synthetic Biology (Ministry of Education), Key Laboratory of Systems Bioengineering (Ministry of Education), SynBio Research Platform, Collaborative Innovation Center of Chemical Science and Engineering (Tianjin), School of Chemical Engineering and Technology, Tianjin University, Tianjin, China; ^2^ Key Laboratory of System Microbial Biotechnology, Tianjin Institute of Industrial Biotechnology, Chinese Academy of Sciences, Tianjin, China

**Keywords:** 5-aminolevulinic acid, biosynthetic pathway, metabolic engineering, detection, application

## Abstract

5-Aminolevulinic acid (5-ALA) is the key precursor for the biosynthesis of tetrapyrrole compounds, with wide applications in medicine, agriculture and other burgeoning fields. Because of its potential applications and disadvantages of chemical synthesis, alternative biotechnological methods have drawn increasing attention. In this review, the recent progress in biosynthetic pathways and regulatory mechanisms of 5-ALA synthesis in biological hosts are summarized. The research progress on 5-ALA biosynthesis via the C4/C5 pathway in microbial cells is emphasized, and the corresponding biotechnological design strategies are highlighted and discussed in detail. In addition, the detection methods and applications of 5-ALA are also reviewed. Finally, perspectives on potential strategies for improving the biosynthesis of 5-ALA and understanding the related mechanisms to further promote its industrial application are conceived and proposed.

## 1 Introduction

5-Aminolevulinic acid (5-ALA), also known as δ-aminolevulinic acid, is an oxygen- and nitrogen-containing hydrocarbon. It is the common precursor of all tetrapyrrole compounds, including chlorophyll, heme and vitamin B_12_ (Kang et al., 2012). It can be synthesized by plants, animals, bacteria and fungi. There are two biosynthetic pathways of it in nature, C4 pathway and C5 pathway respectively. Since it is an endogenous substance that is non-toxic to humans and animals and is easily degraded in the environment without residues, 5-ALA has received widespread attentions in recent years. 5-ALA can be converted into protoporphyrin IX, which is a powerful photosensitizer to cause photosensitive effect (Wang X. F. et al., 2020). Therefore, 5-ALA is successfully used in the treatment of tumors and others diseases (Millesi et al., 2016; Pepa et al., 2020; Suero Molina et al., 2021; Ubbink et al., 2021). 5-ALA is also used as an animal feed additive to improve iron status and immune response in livestock (Wang et al., 2009; Hendawy et al., 2019; Direkbusarakom et al., 2021). It also has functions in higher plants, such as stimulating physiochemical processes, regulating plant growth and development in seed germination, vegetative growth and fruit coloring (Wu et al., 2018). With the continuous discovery of biological functions of 5-ALA, its synthesis and related regulatory mechanisms have attracted increasing attention, which led to further research and progress.

Compared with chemical methods, the biosynthesis of 5-ALA has many advantages as green, sustainable, renewable, and inexpensive technology (Kang et al., 2012). Researchers have made major breakthroughs in the study of synthesis mechanisms of 5-ALA and fermentation process optimization for its biosynthesis over the past 20 years. A number of different microbial hosts were used to produce 5-ALA, including *Escherichia coli* (Zhao and Zhai, 2019), *Corynebacterium glutamicum* (Feng et al., 2016) and *Saccharomyces cerevisiae* (Hara et al., 2019). Recently, the highest titer of biosynthetic 5-ALA reached 18.5 g/L in *C. glutamicum*, which was achieved via evaluation of 5-ALA synthetases from different sources, regulating intracellular activities of 5-ALA synthetase and phosphoenolpyruvate carboxylase, and optimization of the fermentation medium (Chen et al., 2020). In the process of microbial 5-ALA production, the optimization strategies of biosynthetic pathways or metabolic networks based on genome-scale metabolic network models also play an important role in the biosynthesis of 5-ALA by solving the problems of precursor supply (Ren et al., 2018; Hara et al., 2019), proper expression of 5-ALA synthase (Yu et al., 2019; Chen et al., 2020), and regulating the expression levels of key enzymes in downstream pathways (Zhou et al., 2019; Sakurai et al., 2021). Additionally, the detection technologies applied for different samples have also made great progress (Hara et al., 2019; Ren et al., 2018; Tan Z. J. et al., 2019). Previous applications and synthetic methods have been reviewed in detail before in earlier (Kang et al., 2012; Liu et al., 2014; Kang et al., 2017). However, a review focusing on the metabolic engineering strategies to improve 5-ALA production and systematic summary of detection methods with recent advances has not been reported. Therefore, it is of great significance to provide a comprehensive review on 5-ALA for its further synthesis, detection and application.

In this paper, we summarized the recent progress in the research on the biosynthetic pathways and regulatory mechanisms of 5-ALA. The research progress on 5-ALA synthesis in microbial cells is emphasized, and design strategies for obtaining efficient production hosts are analyzed and discussed in detail. Additionally, 5-ALA detection methods as well as its applications in agriculture, medicine and food additives are also reviewed. Finally, possible solutions are proposed to increase our understanding of the regulatory mechanisms and biosynthesis of 5-ALA.

## 2 Biosynthesis and Molecular Regulation of 5-Aminolevulinic Acid

In 1953, 5-ALA was firstly discovered in duck blood (Shemin and Russell, 1953). In the following years, more and more studies proved the existence of 5-ALA synthesis in many animals and higher plants (Harel and Klein, 1972; Samuel and Paul, 1974). Although many efforts have been made to improve the yield of 5-ALA (Ano et al., 1999; Hama and Hase, 1978), its yield was still very low in plants. Some microorganisms in nature also have the ability to synthesize 5-ALA, such as photosynthetic bacteria, which were the first host organisms to synthesize 5-ALA (Sasaki et al., 1987). It is undeniable that some photosynthetic bacteria liking *Rhodobacter sphaeroides* show significant advantages on yield compared to animals and plants (Kamiyama et al., 2000), but its strict requirement demand on fermentation conditions are not conducive to subsequent large-scale industrial production. Therefore, the development of engineered strains for directional produce 5-ALA is in the core of the research in 5-ALA biosynthesis. At present, *Corynebacterium glutamicum*, *Escherichia coli* and *Saccharomyces cerevisiae* are the most commonly used strains for biosynthesis of 5-ALA by metabolic engineering strategies. Additionally, *Streptomyces coelicolor*, *Propionibacterium acidipropionici* (Kiatpapan et al., 2011), *Shewanella oneidensis* (Yi and Ng, 2021) and *Bacillus subtilis* (Liu et al., 2020) have also been attempted to synthesis 5-ALA recently (Supplementary Table S1).

### 2.1 Biosynthetic Pathway of 5-Aminolevulinic Acid

**FIGURE 1:**
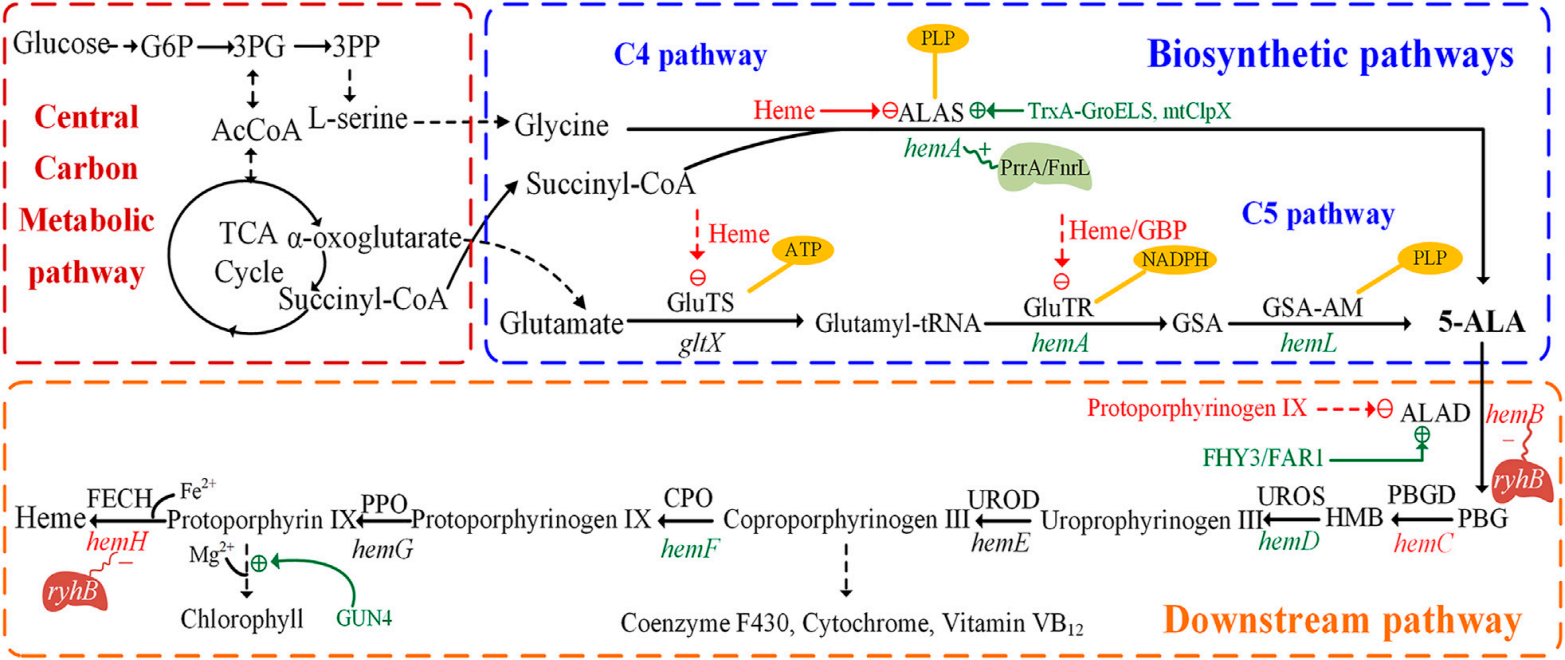
Biosynthetic pathways and downstream pathways of 5-aminolevulinic acid. The figure is divided into three parts: central carbon metabolic pathways, biosynthetic pathways and downstream pathways. The dashed green line indicates positive regulation, the dashed red line indicates feedback inhibition. The genes in green or red represent the enzymes that are positive or negative for 5-aminolevulinic acid accumulation, respectively. The polygons represent transcriptional regulators and red or blue represent positive/negative regulation. PBGD, porphobilinogen deaminase; UROS, uroporphyrinogen III synthase; UROD, uroporphyrinogen decarboxylase; CPO, coproporphyrinogen oxidase; PPO, protoporphyrinogen oxidase; FECH, ferrochelatase; FHY3, Far-red Elongated Hypocotyl 3; FAR1, Far-red Impaired Response 1; mitochondrial ClpX (mtClpX); 3PG, 3-phosphoglycerate.

5-ALA was confirmed as the common precursor of tetrapyrrole compounds and was found in many organisms. Two natural 5-ALA biosynthesis pathways are known to date. One is the C4 pathway (Shemin pathway), which was firstly reported by Shemin and Russell (1953). It exists in animals, yeast, some protozoa and purple non-sulfur photosynthetic bacteria. In this pathway, glycine and succinyl-CoA are condensed to 5-ALA under the catalysis of 5-aminolevulinic acid synthase (ALAS), with pyridoxal 5′-phosphate (PLP) as the cofactor (Figure 1) (Sasaki et al., 2002). Succinyl-CoA is synthesized by methylmalonyl-CoA mutase, which utilizes vitamin B_12_ as an essential co-factor (38). The other is the C5 pathway (Beale pathway), which starts from the discovery of 5-ALA in *Chlorella vulgaris* by Beale (Beale, 1970). Glutamate produced via the TCA cycle acts as the substrate of the C5 pathway (Kang et al., 2012). The C5 pathway is mainly present in higher plants, algae, and many bacteria. The pathway starts with the ligation of tRNA and glutamate to generate L-glutamyl–tRNA, catalyzed by glutamyl–tRNA synthetase (GluTS) (Figure 1). The NADPH-dependent glutamyl-tRNA reductase (GluTR) reduces the carboxyl group of Glu–tRNA to a formyl group, which enables the conversion of L-Glu–tRNA into L-glutamic acid 1-semialdehyde (GSA). In the last step, 5-ALA is created through transamination by glutamate-1-semialdehyde aminotransferase (GSA-AM) (Moser et al., 2001). The enzymes involved in this pathway, GluTS, GluTR and GSA-AM, are encoded by the genes *gltX*, *hemA* (homonymic with the ALAS gene of the C4 pathway) and *hemL*, respectively. However, a few microorganisms have both C4 and C5 pathways with *Euglena gracilis* being a well-known example (Weinstein and Beale, 1983).

### 2.2 Regulation of 5-Aminolevulinic Acid Biosynthesis

#### 2.2.1 Main Regulation Mechanism of the C4 Pathway

In the C4 pathway, 5-ALA is formed by ALAS, encoded by the *hemA* or *hemT* gene, which catalyzes the condensation of succinyl-CoA and glycine. The *hemA* gene provides most of the ALAS activity, much more than *hemT* (Neidle and Kaplan, 1993; Zeilstra-Ryalls and Kaplan, 1995). ALAS is a rate-limiting enzyme for 5-ALA biosynthesis, and the synthesis of this enzyme is itself highly regulated via feedback regulation of the *hemA* and *hemT* genes (Sasaki et al., 1995). The transcription of *hemA* from its two promoters is regulated by the DNA-binding proteins FnrL and PrrA in *R. sphaeroides* (Figure 1), which might be the only transcription factors involved in the oxygen responsiveness of *hemA* (Fales et al., 2002; Ranson-Olson and Zeilstra-Ryalls, 2008). Furthermore, the enzymatic activity of ALAS is subject to feedback inhibition by the end-product heme (Tian et al., 2011). Another important protein, mitochondrial ClpX, directly stimulates 5-ALA synthase in *S. cerevisiae* by catalyzing the incorporation of its cofactor, pyridoxal 5’ phosphate (Kardon et al., 2020).

#### 2.2.2 Main Regulation Mechanism of the C5 Pathway

In the C5 pathway, 5-ALA is derived from a transfer RNA (tRNA)-bound glutamate in three enzymatic steps. In some bacteria, the GluTS complexed with ATP and tRNA^Glu^, as well as its five domains for substrate or cofactor recognition (Zn^2+^, Mg^2+^, ATP, glutamate and tRNA) and catalytic activity have been studied (Dubois et al., 2009). In *E. coli*, GluTS is responsible for the formation of glutamyl-tRNA and 5-ALA synthesis (Beale et al., 1975). The regulation of GluTS activity is multi-dimensional and complex. Its activity is regulated by intracellular heme levels (Levican et al., 2007) (Figure 1). GluTR was proved to be the key enzyme of the C5 pathway, forming a tight complex with GSA-AM to protect the highly reactive intermediate GSA-AM (Moser et al., 2001). The activated glutamate is converted to GSA by the NADPH dependent GluTR enzyme, which in turn is quickly converted to 5-ALA by GSA-AM. In addition, GluTR is regulated at the posttranscriptional and protein levels (Woodard and Dailey, 1995; Wang et al., 1999). When the concentration of heme is sufficient, the activity of GluTR is strongly inhibited to reduce the synthesis of 5-ALA. Furthermore, the complex of heme and GluTR-binding protein also inhibits the interaction between GBP and GluTR, causing the deregulation of GluTR (Richter et al., 2019).

Similar but more complex regulation mechanisms also exist in higher plants. Feedback inhibition plays an important regulative role in 5-ALA synthesis via the C5 pathway. It was reported that GluTR is the target of FLU protein in higher plants (Zhang M. et al., 2015). The negative regulator, FLU protein, has been proposed to have a synergetic role in the chlorophyll branch, similar to the function of heme (Kauss et al., 2012). The transcription factors like Far-red Elongated Hypocotyl 3 (FHY3) and Far-red Impaired Response 1 (FAR1) have a positive regulative role in chlorophyll biosynthesis since they can bind and activate the expression of *hemB* (encodes ALAD), thereby upregulating the synthesis of 5-ALA in higher plants (Tang et al., 2012).

## 3 Metabolic Engineering Strategies for 5-Aminolevulinic Acid Biosynthesis

**FIGURE 2:**
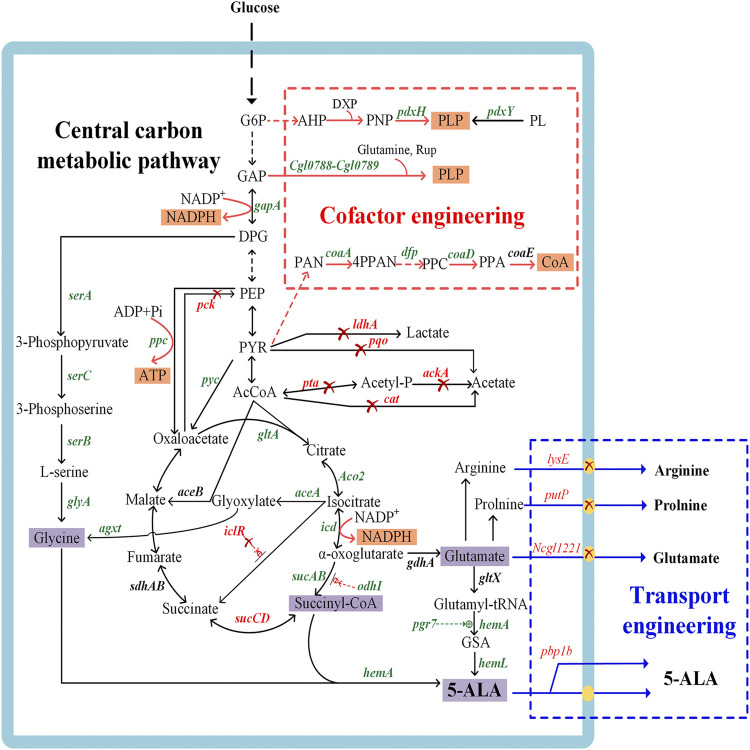
Genetic manipulations in metabolic engineering strategies for 5-aminolevulinic acid biosynthesis. The genes in green or red represent the enzymes that should be overexpressed or inactivated to accelerate 5-ALA accumulation, respectively. The red cross indicates that the pathways are disrupted, the dashed green line indicates positive regulation, and the dashed red line indicates feedback inhibition. *pdxH*, encoding pyridoxal 5-phosphate synthase; *pdxY*, pyridoxal kinase; *Cgl0788-Cgl0789*, pyridoxal 5′-phosphate synthase gene; *gapA*, encoding glyceraldehyde 3-phosphate dehydrogenase; *serA*, encoding 3-phosphoglycerate dehydrogenase; *serB*, encoding phosphoserine phosphatase; *serC*, encoding phosphoserine aminotransferase; *glyA*, encoding serine hydroxymethyl transferase; *coaA*, encoding pantothenate kinase; *dfp*, encoding dephospho-CoA kinase; *coaD*, encoding pantetheine-phosphate adenylyltransferase; *coaE*, encoding dephospho-CoA kinase; *ppc*, encoding phosphoenolpyruvate carboxylase; *pyc*, encoding pyruvate carboxylase; *pck*, encoding phosphoenolpyruvate carboxykinase; *gltA*, encoding citrate synthase; *ldhA*, encoding L-lactate dehydrogenase; *pqo*, encoding pyruvate:menaquinone oxidoreductase; *pta*, encoding phosphotransacetylase; *ackA*, encoding acetate kinase; *cat*, encoding acetyl-CoA:CoA transferase; *ACO2*, encoding aconitase; *icd*, encoding isocitrate dehydrogenase; *sucAB*, encoding α-oxoglutarate dehydrogenase; *odhI*, encoding α-oxoglutarate dehydrogenase inhibitor; *sucCD*, encoding succinyl-CoA synthetase; *aceA*, encoding isocitrate lyase; *aceB,* encoding malate synthase; *iclR*, encoding the transcriptional regulator of glyoxylate cycle genes *aceBAK*; *gdhA*, encoding glutamate dehydrogenase; *gltX*, encoding glutamyl-tRNA synthetase; *hemA*, encoding glutamyl-tRNA reductase; *hemL*, encoding glutamate-1-semialdehyde aminotransferase; *pgr7*, encoding *hem1* stimulator protein; *hemA*, encoding 5-aminolevulinate synthase; *rhtA*, encoding serine/threonine transporter; *lysE*, encoding lysine/arginine transporter; *putP*, encoding L-proline transporter; *Ncgl1221*, encoding glutamate transporter; *agxt*, encoding glyoxylate aminotransferase from *Homo sapiens*. G6P, glucose-6-phosphate; 3PP, 3-phosphoserine; GAP, glyceraldehyde 3-phosphate; DPG, 1,3-bisphosphoglyceric acid; PEP, phosphoenolpyruvate; PYR, pyruvic acid; AcCoA, acetyl-CoA; AHP, 3-hydroxy-1-aminoacetone phosphate; DXP, deoxyxylulose 5-phosphate; PNP, pyridoxine 5′-phosphate; PL, pyridoxal; Rup, ribulose 5-phosphate; PAN, pantothenate; 4PPAN, 4′-phosphopantothenate; PPC, 4′-phosphopantethenine; PPA, dephpspho-CoA; CoA, coenzyme A; ADK, adenylate kinase; HK, hexokinase; ZWF, glucose-6-phosphate dehydrogenase; PGL, phosphogluconolactonase; GND, 6-phosphogluconate dehydrogenase; R5P, D-ribulose 5-phosphate.

A number of metabolic engineering strategies have been developed with the aim to establish an industrially sustainable biosynthesis route for 5-ALA (Figure 2), including engineering of key enzymes, redistribution of central carbon fluxes toward precursors, blockage of downstream pathways, cofactor engineering, and transporter engineering. The recent research on improving 5-ALA biosynthesis is summarized in Supplementary Table S1.

### 3.1 Engineering of Key Enzymes for 5-Aminolevulinic Acid Biosynthesis

#### 3.1.1 5-Aminolevulinate Synthase in the C4 Pathway

ALAS is the key enzyme for 5-ALA synthesis via the C4 pathway, and its isoenzymes are encoded by different redundant genes (Kang et al., 2012). Tai et al. (1988) identified the two genes *hemA* and *hemT* as potentially encoding ALAS in *R. sphaeroides*, which was further confirmed by Neidle et al., as well as Neidle and Kaplan (1993). Compared with the *hemT* gene, the expression of *hemA* is more sensitive to feedback inhibition by heme in wild-type *R. sphaeroides* 2.4.9 (Stoian et al., 2018). When the expression of *hemA* is inhibited, *hemT* starts to play an alternative role in 5-ALA synthesis. In addition, *hemO* is another redundant gene that also encodes ALAS and can support the synthesis of 5-ALA in *R. sphaeroides* (Zhang et al., 2013). Engineered *E. coli* overexpressing the *hemO* gene produced 6.3 g/L of 5-ALA, which was higher than the 5.7 g/L of the strain overexpressing *hemA* (Zhang et al., 2013). Moreover, both ALAS activity and its affinity for substrates in the host cell influence the synthesis of 5-ALA. For example, overexpression of the *hemA* gene from *R. palustris* ATCC 17001 yielded a 5-ALA titer of 3.8 g/L, which was 15.2% and 18.9% higher than those produced by two other ALAS genes in *C. glutamicum* (Chen et al., 2020).

To decrease the feedback inhibition by heme and increase the thermostability of ALAS, the enzyme was modified using different strategies. For example, the H29R and H15K ALAS mutants were screened from several single variants by computer-assisted rational design (Tan Z. J. et al., 2019). The 5-ALA levels of *C. glutamicum* strains expressing the mutants respectively increased by 6 and 22 compared with the strain expressing wild-type ALAS (Tan Z. J. et al., 2019)*.* To enhance the soluble protein expression, Yu et al. (2019) used TrxA and its chaperone GroELS to improve the activity of ALAS (Figure 1). Recently, Yu et al. (2022) further biosynthesized 5-ALA by integrating and co-expressing *groELS* and *hemA* in chromosome. The final 5-ALA titer increased to 15.6 g/L after supplying ferric ion and optimizing the glucose-glycerol as a mixed carbon source. In addition, the transcription of *hemA* was found to be influenced by changes of the cellular redox state (Zeilstra-Ryalls and Kaplan, 1996). Most importantly, the expression strength of ALAS should be maintained at a moderate level in case of an imbalance between 5-ALA synthesis and the TCA cycle (Chen et al., 2020). In conclusion, reliving the complex feedback inhibition of ALAS and maintaining moderate expression levels are the key to achieving high productivity of 5-ALA.

#### 3.1.2 Glutamyl-tRNA Reductase and Glutamate-1-semialdehyde Aminotransferase in the C5 Pathway

**FIGURE 3:**
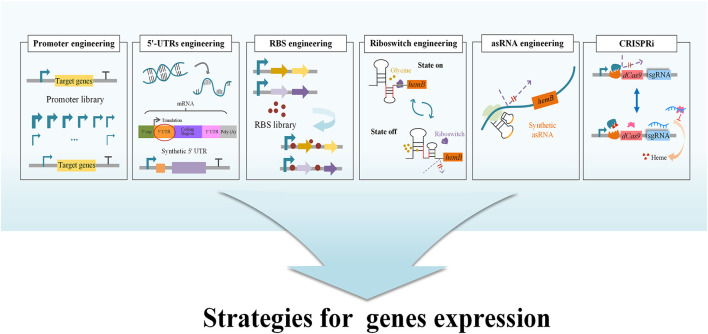
Strategies for tuning the expression and regulation of genes. Tuning gene expression and regulation requires a balance between cell growth and a specific biological activity in order to transform the original system within the microbial cells into one that generates the target product. The strategies include several methods, such as CRISPRi, Riboswitch engineering, Synthetic asRNA engineering, RBS engineering. Promoter engineering, 5′-UTR engineering and RBS library can be used to optimize the expression of the target genes, and thereby increase the biosynthesis of 5-ALA. In riboswitch engineering, a glycine-OFF riboswitch was utilized to dynamically downregulate ALAD expression in the presence of glycine, while small amounts of glycine allow *hemB* to be expressed normally (Zhou et al., 2019). Using CRISPRi technology, a heme-responsive regulatory system was developed to control the concentration of heme dynamically and precisely. At low heme concentration, the regulatory system hinders the expression of CRISPRi (Zhang et al., 2020a).

GluTR (encoded by *hemA*) and GSA-AM (encoded by *hemL*) are two charging enzymes in the C5 pathway (Figure 2). GluTR is the key enzyme forming a tight complex with GSA-AM to protect the highly reactive intermediate GSA-AM (Moser et al., 2001). GluTR activity is also inhibited by heme and further influences the synthesis of 5-ALA. However, it has been reported that a C170A mutant of HemA from *Salmonella enterica* is not influenced by heme (Jones and Elliott, 2010). Although this work was a breakthrough in eliminating feedback inhibition from heme, the influence of the C170A mutant to 5-ALA synthesis was still unknown. Subsequently, HemA mutants from different sources with higher activity and stability were screened to increase the production of 5-ALA combined with HemL in *E. coli* and *C. glutamicum* (Kang et al., 2011a; Yu et al., 2015; Zhang et al., 2017; Ko et al., 2019). To increase the expression level of the two genes *hemA* and *hemL*, different metabolic engineering strategies were applied with good results, including promoter engineering (Noh et al., 2017), transcriptional regulation with synthetic 5′-untranslated regions (5′-UTRs) (Noh et al., 2017) and RBS engineering (Zhang and Ye, 2018; Zhang J. L. et al., 2019) (Figure 3). Recently, the engineered strain *E. coli* Transetta GTR/GBP co-overexpressing the stimulator protein GBP (encoded by *pgr7*) could produce 3.08 g/L of 5-ALA, representing a 2.37-increase compared to overexpression of *hemA1* alone (Zhao and Zhai, 2019). In general, overexpression of the two genes *hemA* and *hemL* both contributes to the biosynthesis of 5-ALA. Further exploring the mechanisms that control these two genes and reducing other influencing factors are effective strategies for increasing the production of 5-ALA.

### 3.2 Enhancing Precursor Supply by Redistributing the Fluxes of Central Carbon Metabolism

#### 3.2.1 Metabolic Engineering of Central Carbon Metabolic Pathways for Supplying Precursors in the C4 Pathway

5-ALA is produced by the condensation of succinyl-CoA and glycine in the C4 pathway (Figure 2). Previous studies found that addition of the precursors succinyl-CoA and glycine was conducive to enhancing the production of 5-ALA (Choi et al., 1999; Liu et al., 2010), which indicated that the supply of these two precursors might be the main bottleneck of 5-ALA synthesis. Consequently, many metabolic engineering studies have been undertaken in different microorganisms to increase the supply of these precursors (Figure 2). Further advances will make it possible to directly regulate the metabolic flux toward succinyl-CoA and glycine.

There are two main strategies to improve the intracellular succinyl-CoA pool. One relies on reinforcing the carbon flux toward the TCA cycle. For example, overexpression of *gltA* (encoding citrate synthase), *ppc* (encoding phosphoenolpyruvate carboxylase) and *pyc* (encoding pyruvate carboxylase), together with the deletion of *pck* (encoding phosphoenolpyruvate carboxykinase), can redirect the carbon flux towards the TCA cycle (Feng et al., 2016). Combined with the knockout of by-product pathways that produce acetate and lactate, the production of 5-ALA by engineered *C. glutamicum* was further increased from 1.44 to 2.07 g/L (Feng et al., 2016). However, high overexpression levels of target genes in central carbon metabolic pathways might interfere with the synthesis of 5-ALA and destroy the balance between complicated pathways. Recently, overexpression of *ppc* resulted in decreased accumulation of 5-ALA while an obvious elevation of production (34.1% higher than control) could be achieved by finer tuning using a moderate RBS (Chen et al., 2020). Another approach relies on strengthening the metabolic flux from the TCA cycle to the precursor of 5-ALA synthesis. Succinyl-CoA synthetase, encoded by *sucCD*, catalyzes the reversible reaction between succinate and succinyl-CoA. The inactivation of *sucCD* can redirect the carbon flux toward the 5-ALA synthesis branch rather than the reductive TCA cycle (Yang et al., 2016; Ding et al., 2017). And cutting down glutamate biosynthesis pathway (encoded by *gdhA*) increased the flux from α-oxoglutarate to succinyl-CoA, which increased the 5-ALA titer 2-fold compared to the control (Ge F. et al., 2021). Apart from preventing the flux to the reductive TCA cycle, reinforcing the gene expression in the oxidative arm of the TCA cycle is equally significant for the synthesis 5-ALA. For example, the overexpression of *ACO2* (encoding aconitase) in the oxidative-arm of the TCA cycle could enhance the 5-ALA yield in *S. cerevisiae* (Hara et al., 2019). In *C. glutamicum* CgS1, overexpressing α-oxoglutarate dehydrogenase (encoded by *sucAB*) caused a 6% increase of the 5-ALA titer, while isocitrate dehydrogenase (encoded by *icd*) had a negative effect in this case (Yang et al., 2016). These results indicated that the regulation of multiple genes in the TCA cycle is relatively complex, and a design based on genome-scale metabolic network model is a more effective approach for increasing 5-ALA production.

Glycine is another precursor in the C4 pathway and it limits the yield of 5-ALA. The supply of glycine is a well-known bottleneck for the high-level production of 5-ALA, and this problem is mainly solved by adding exogenous glycine in the fermentation process (Fu et al., 2010; Kang et al., 2010; Kang et al., 2011b). Some experts also attempted to provide glycine for 5-ALA biosynthesis via metabolic engineering strategies (Figure 2). 3-phosphoglycerate dehydrogenase (encoded by *serA*) is the first enzyme of the glycine synthesis pathway and is tightly regulated via feedback inhibition by L-serine analogues, which could be released by mutations (Ding et al., 2017). The level of 5-ALA in a *C. glutamicum* strain overexpressing *serA* ^*Δ197*^ *, serB* and *serC* increased by 70% compared to the parental strain with an unmodified glycine synthesis pathway (Zou et al., 2017). Recently, an unnatural pathway for the production of 5-ALA was construct by introducing the *agxt* gene from *Homo sapiens* to transform glyoxylate into glycine and finally realize 5-ALA synthesis without glycine addition (Ren et al., 2018). The proposed new approach is particularly useful to circumvent the problem of precursor supply, which was difficult to be engineered. In addition, our own research demonstrated the moderate amounts of glycine indeed improve 5-ALA synthesis (Zou et al., 2017), while excessive glycine poisons the host cells and influences the final yield (Sasaki et al., 2002; Fu et al., 2010). Therefore, it is necessary to continuously maintain an appropriate concentration of glycine in order to realize high productivity of 5-ALA.

#### 3.2.2 Metabolic Engineering of Central Carbon Metabolic Pathways for Providing Precursors to the C5 Pathway

Glutamate is the precursor of the C5 pathway, mainly produced from α-oxoglutarate by glutamate dehydrogenase (GS) (encoded by *gdhA*). To improve the supply of glutamate, the carbon flux from the TCA cycle or glyoxylate cycle is redistributing towards α-oxoglutarate (Figure 2). The carbon flux of the TCA cycle was diverted to glutamate by overexpressing the *gltA* gene, deleting *sucA* (encoding α-oxoglutarate dehydrogenase), and precisely regulating the glyoxylate cycle using an appropriate transcription strength of *aceA* (encoding isocitrate lyase) in an engineered *E. coli* overexpressing *hemA* ^*M*^ and *hemL*, generating the flux-optimized strain WSAL4 (Noh et al., 2017). The level of 5-ALA was increased 3.6-fold compared to the control strain. In addition, repressing the transformation of α-oxoglutarate to succinyl-CoA and overexpressing an oxoglutarate dehydrogenase inhibitor (OdhI) reinforced the supply of glutamate in *C. glutamicum* (Ko et al., 2019). Double-mutated OdhI with eliminated phosphorylation and inhibited oxoglutarate dehydrogenase activity was further used to synthesize 5-ALA.The 5-ALA production of the double-mutated OdhI strain increased to 2.1 g/L, which was much higher than the 0.35 g/L of the strain expressing wild-type OdhI (Ko et al., 2019). Moreover, Zhang et al. dynamically regulated the expression of *odhA* (α-ketoglutarate dehydrogenase complex E1 subunit gene) with the strategies of auto-inducible metabolic engineering by a growth-regulated promoter P_*CP_2836*_, which increased the production of 5-ALA by 1-fold (Zhang C. L. et al., 2020).

Repressing glutamate degradation and transport is also an alternative strategy. Recent studies reported that inactivation of a glutamate-transporter membrane protein (encoded by *Ncgl1221*) could drastically decrease the secretion of glutamate (Zhang and Ye, 2018) and promote the accumulation of intracellular 5-ALA in *C. glutamicum* (Chen et al., 2015; Zhang and Ye, 2018) (Figure 2). There are also other glutamate transport proteins, such as GltP, GltJ, GltK, GltL and GltS, which are strongly related to the intracellular glutamate concentration (Zhao and Zhai, 2019). Moreover, deleting the transport proteins of the downstream by-products of glutamate, proline and arginine also promoted the synthesis for 5-ALA (Zhang and Ye, 2018).

### 3.3 Regulated Expression of Key Genes in Downstream Pathways

Tetrapyrrole compounds are produced from 5-ALA in specific downstream pathways and are important for physiological and metabolic activities of organisms. The expression levels of downstream genes affect enzymes activity and transcription of the 5-ALA biosynthesis pathway as well as cell growth. In other words, regulated expression of key genes in downstream pathways is conducive to further increase 5-ALA production.

ALAD is the first enzyme in the biosynthetic pathways of heme, chlorophyll and other essential porphyrins. They all start from 5-ALA in the downstream pathway and ALAD has a close relationship with final accumulation of 5-ALA. The metabolic intermediate protoporphyrinogen IX inhibits the expression of ALAD and further influences the accumulation of 5-ALA (Figure 1) (Zhang J. L. et al., 2015; Zhang et al., 2016). Many studies have attempted to improve the concentration of 5-ALA by inhibiting the expression of ALAD to reduce the intracellular utilization of 5-ALA (Ding et al., 2017; Yu et al., 2015). As ALAD is an important enzyme catalyzing the first step in the synthesis of many essential porphyrins, the complete loss of ALAD activity has a negative impact on cell growth. The replacements of the initiation codon or promoter to regulate ALAD expression at the transcriptional and translational levels is another validated measure to realize the balance between cell growth and 5-ALA biosynthesis. After altering the most preferred initiation codon AUG into GUG in *E. coli*, the production of 5-ALA significantly increased from 1.31 to 1.76 g/L (Ding et al., 2017). Promoter engineering is another effective way to directly downregulate ALAD expression at the transcriptional level by replacing its promoter with constitutive promoters and stationary-phase promoters (Figure 3). A strain in which the stationary-phase promoter *fliCp* was introduced displayed superior performance, and the final production of 5-ALA was 2.68 g/L (increased by 11% over the control strain) (Zhang J. L. et al., 2019). Additionally, a glycine-OFF riboswitch from *Clostridium pasteurianum*, Apt2#82, was utilized to dynamically downregulate ALAD expression in the presence of glycine, resulting in an 11% improvement of 5-ALA production in recombinant *E. coli* (Zhou et al., 2019) (Figure 3). It is reported that the antisense RNA (asRNAs) of *hemB* could decrease its’ mRNA level by 50%, and the production of 5-ALA was increased by 17.6% (Ge F. L. et al., 2021) (Figure 3). In a recent research, CRISPR interference (CRISPRi) was used to repress the expression of *hemB*, and the titer of 5-ALA increased 3.7 fold compared to the control (Miscevic et al., 2021). However, weakening the activity of ALAD cannot guarantee the elevation of production (Xie et al., 2003; Zhang J. L. et al., 2019), implying that ALAD has an appropriate range of activity and advanced research should be carried on.

The combinatorial perturbation of multiple genes in the downstream pathway has attracted widespread attention, with most studies focusing on engineered *E. coli*. Among several genes in the downstream pathway, the upregulation of *hemD* and *hemF* facilitated the accumulation of 5-ALA, while the overexpression of *hemB*, *hemG* and *hemH* had no effect (Zhang J. L. et al., 2019). The expression of *hemD* and *hemF* with moderate intensity, as well as overexpression of *hemA* and *hemL* increased the production of 5-ALA in recombinant *E. coli* LADF-6 to 3.25 g/L, 3.78-fold higher than the parental strain (Zhang J. L. et al., 2015). Recent studies indicated that the iron regulator small RNA *ryhB* distinctly downregulates the transcription of *hemB* and *hemH*, perturbing the accumulation of heme and 5-ALA (Figure 1) (Li et al., 2014). Subsequently, further studies investigated the transcriptional regulatory mechanism of *ryhB* in relation to other genes of the heme synthesis pathway. It was demonstrated that both overexpression of *hemD* and *hemF* downregulated the transcription of *ryhB*, while overexpression of *hemB* resulted in an unexpected increase (Zhang et al., 2016). In addition, a heme-responsive regulatory system (Figure 3) was designed and constructed by CRISPRi to dynamically and precisely control the downstream pathways for 5-ALA synthesis (Zhang et al., 2020a). In this work, a H149D mutant was obtained by semi-rational design with site saturated mutagenesis of HrtR from *Lactococcus lactis*, and used as a biosensor to dynamically regulate the expression of *hemC* and *hemH* in the downstream pathways, which resulted in a 5-ALA titer of up to 5.35 g/L 5-ALA in batch-cultures of *E. coli* (Zhang et al., 2020a).

Thus, the biosynthesis of 5-ALA interacts with heme synthesis in downstream pathways and the deeper regulation is complex and ambiguous. Therefore, the simple deletion or overexpression of genes may not be able to produce a high yield of 5-ALA, and more elaborate regulatory strategies need to be developed to achieve a balance between cell growth and 5-ALA biosynthesis.

### 3.4 Cofactor Engineering

The supply of cofactors immediately impacts the production of metabolic products and the modification in cofactor engineering can accelerate the yield of targeted products, such as acetoin and 2,3-butanediol (Yang et al., 2017). During the synthesis of 5-ALA, the conversion of substances and the activation of enzymes mostly relies on cofactors such as pyridoxal PLP, coenzyme A, NADPH/NADP, etc. The supply of sufficient cofactors is of great significance to ensure the flow of electrons, activation of enzymes and maintenance of their stability. Currently, studies on cofactor supply mainly focus on the strengthening of the pyridoxal 5′-phosphate and coenzyme A supply by exogenous addition and metabolic pathway engineering, and also few involves the metabolic engineering of ATP and NAD(P)H (Figure 2).

#### 3.4.1 Pyridoxal 5′-Phosphate

Pyridoxal 5′-phosphate (PLP) is a biologically active form of vitamin B_6_ that can catalyze many enzymatic and non-enzymatic reactions (Heyl et al., 1951; Hayashi et al., 1990). At the same time, PLP is also an essential cofactor involved in both the C4 and C5 pathways. The key residues of these enzymes that interact with PLP are suspected to undergo extensive movements and contribute to the formation and stabilization of the functional conformation at the active sites (Brown et al., 2018). In the C4 pathway, ALAS condenses two precursors into 5-ALA with the assistance of PLP as an important cofactor. Firstly, PLP combines with glycine to form a covalent adduct, and further triggers transaldimination in the microenvironment. Then the PLP-glycine adduct releases CO_2_ to synthesize 5-ALA by decarboxylation or deprotonation with the participation of succinyl-CoA (Kang et al., 2012). The interplay of the PLP cofactor with the protein moiety determines and modulates the multi-intermediate reaction cycle of ALAS (Stojanovski et al., 2019). At the same time, PLP binding induces re-ordering of active sites and key residues to achieve the active conformation of the enzyme according to the crystal structure of ALAS (Brown et al., 2018). GSA-AM is the last enzyme in the C5 pathway and also depends on PMP/PLP to synthesize 5-ALA. In view of structural similarity with ALAS, GSA-AM has been hypothesized to be the evolutionary precursor of ALAS (Schulze et al., 2006). One proposed reaction mechanism supported by kinetic studies posits that PLP is initially present as pyridoxamine 5′-phosphate (PMP) (Pugh et al., 1992). GSA is converted to 5-ALA via the intermediate 4,5-diaminovalerate along with the conversion of PMP into PLP (Li et al., 2018).

It was confirmed that the addition of PLP has a positive effect on 5-ALA production (Zhang J. L. et al., 2019). Subsequently, the supply of PLP was reinforced by slightly strengthening the native PLP pathway, and the titer of 5-ALA was increased by 7% to 2.86 g/L in the C5 pathway (Zhang J. L. et al., 2019). In a recent research, the overexpression of *Cgl0788*-*Cgl0789* operon (encoding pyridoxal 5′-phosphate synthase) in engineered *C. glutamicum* also improved the production of 5-ALA by 15.6% (Yan et al., 2020), which proved that the metabolic modification of PLP pathway promoted the production of 5-ALA (Figure 2). Similarly, PLP is also important in the C4 pathway and removing PLP led to complete loss of enzyme activity (Choi et al., 1999; Liu et al., 2010). Given the obvious efficiency of PLP in the C5 pathway, we speculate that it also may have a significant positive effect on 5-ALA synthesis in the C4 pathway, which is confirmed by a recent research. After integrating the gene encoding pyridoxal kinase (PdxY) into the chromosome and coexpressing ALAS from *R. capsulatus*, the 5-ALA titer of recombinant *E. coil* increased 4.33-fold to 1.99 g/L (Xue et al., 2021).

#### 3.4.2 Coenzyme A

Coenzyme A (CoA) is another essential cofactor in numerous biosynthetic pathways, such as the synthesis of pantothenate (vitamin B_5_), cysteine, and ATP (Takagi et al., 2010). Moreover, CoA has a direct relationship with the synthesis of succinyl-CoA, indicating that perturbation of CoA metabolism might have a distinct influence on 5-ALA.

Pantothenate kinase (encoded by *coaA*) is an essential protein in the CoA biosynthesis pathway and it is regulated via feedback inhibition by CoA (Song and Jackowski, 1992; Rock et al., 2000). The overexpression of *coaA* in recombinant *C. glutamicum* led to a 10% increase of 5-ALA production (Yang et al., 2016). The synthesis of CoA from pantothenate involves a four-step enzymatic process, whereby the reactions catalyzed by pantothenate kinase and phospho-pantetheine adenylyl transferase (encoded by *coaD*) are major rate-limiting steps (Vadali et al., 2004; Lee et al., 2013). The effects of all genes in the CoA synthetic pathway on 5-ALA was confirmed, and it was demonstrated that upregulating all the genes of the CoA synthesis pathway, except for *coaE* (encoding dephospho-CoA kinase), is beneficial to the accumulation of 5-ALA (Ding et al., 2017). The production of 5-ALA reached 1.00 g/L (increased by 45.1%) by co-overexpressing the *coaA* ^*M*^, *dfp* and *coaD* genes (Ding et al., 2017) (Figure 2).

#### 3.4.3 ATP and NAD(P)H

As cofactor, ATP and NAD(P)H directly participate in the transmission of energy and the transport of electrons to influence the yield of 5-ALA (Ren et al., 2018). The strains are capable of synthesizing these cofactors, while their own regeneration is generally insufficient for the overproduction of 5-ALA. Accounting for the importance of cofactors for growth and production, it was assumed that enhancing cofactor regeneration pathways may facilitate increased 5-ALA production. In the biosynthesis of 5-ALA in the C5 pathway, the reactions catalyzed by GluTS and GluTR are respectively ATP and NADPH dependent (Figure 1). The appropriate overexpression of phosphoenolpyruvate (PEP) carboxykinase (PCK, encoded by *pckA*) from *Mannheiemia succiniciproducens*, which can irreversibly convert PEP to oxaloacetate and generates ATP, increased the production of 5-ALA by 12.7% (Zhang C. L. et al., 2020). In order to improve the pool of NADPH, isocitrate dehydrogenase (IDH, encoded by *icd*) and NADP^+^- dependent glyceraldehyde 3-phosphate dehydrogenase (GAPDH, encoded by *gapA*) were successively overexpressed, and the production of 5-ALA respectively increased 13.5% and 26.7% compared with the control (Zhang C. L. et al., 2020) (Figure 2). Although few research concerned on increasing ATP flux by metabolic engineering strategies, the potential positive effect on increasing the yield of 5-ALA deserves further attention from researchers in the future.

### 3.5 Accelerated Transport of Intracellular 5-Aminolevulinic Acid

High-level synthesis of target products may be harmful to the microbial host cells and further inhibit the production of the target product to some extent. 5-ALA is one of the typical representatives, which easily result in the accumulation of reactive oxygen species (ROS) that has toxic effects on cells. It is therefore critical to improve 5-ALA production by accelerating intracellular 5-ALA transfer to the extracellular environment, alleviating the cytotoxicity.

Because its physical properties are close to those of proteinogenic amino acids, different transport proteins with the ability to transport amino acids and their analogues have been used to improve the production of 5-ALA (Figure 2) (Moser et al., 2001). The serine/threonine transporter RhtA has been widely used in numerous recombinant strains and exhibited excellent results in increasing the extracellular 5-ALA concentration. Kang et al. firstly applied the *rhtA* gene in 5-ALA synthesis and successfully demonstrated its feasibility for further improvement of production (Kang et al., 2011a). Recently, overexpressing *rhtA* with a T7 system composed of orthogonal T7RNA polymerase and the T7 promoter increased 5-ALA production 40-fold in *E. coli* (Tan S. I. et al., 2019). Introduction of *rhtA* into the high-yield recombinant *C. glutamicum* AL-OI** increased production of 5-ALA by 28% over the control (Ko et al., 2019). Interestingly, the expression level of *rhtA* was upregulated 17-fold in the high-yield *E. coli* Transetta GTR/GBP co-expressing *HemA1* and *pgr7* from *A. thaliana* (Zhao and Zhai, 2019). The timely and effective transport of 5-ALA outside the cell is of great significance for the continuous synthesis of 5-ALA. It is undeniable that RhtA is not the only protein that can transport 5-ALA. However, only few export proteins have been found, and the discovery of transporters remains a great challenge.

A second strategy for facilitating the transport of 5-ALA relies on changing the permeability of the cell wall. Penicillin inhibits the synthesis of cell wall components by restraining the activity of penicillin-binding proteins and further benefits the accumulation of 5-ALA (Laneelle et al., 2013; Ramzi et al., 2015). However, the exogenous addition of penicillin not only increases the production cost but is also not environmentally friendly. Our previous study directly influenced the synthesis of the cell wall by deleting the non-essential high molecular-weight penicillin-binding protein genes *pbp1a*, *pbp1b*, and *pbp2b* individually, and all were positive for the production of 5-ALA with increases of 13.53, 29.47, and 22.22%, respectively (Feng et al., 2016), demonstrating the vital role of cell wall permeability for extracellular 5-ALA accumulation.

### 3.6 Cell-free Metabolic Engineering *in vitro*

The production of 5-ALA *in vivo* is limited by low volumetric productivity, byproduct losses and complex fermentation processes. Moreover, the producer strains tend to be exposed to the toxic effects of ROS, which affects the normal growth of the cells (Zeng et al., 2015). To address these limitations, cell-free metabolic engineering that utilizes purified enzymes or crude cell extracts to synthesize 5-ALA was proposed (Dudley et al., 2015).

In an early study using cell-free extracts of recombinant *E. coli* with the addition of succinate, glycine, LA and ATP, the production of 5-ALA reached 22.6 mM (2.96 g/L) *in vitro* (Van Der Werf and Zeikus, 1996). By contrast, the same recombinant strain from which the cell extract was prepared was only able to produce 2.25 mM (0.30 g/L) 5-ALA *in vivo*, which illustrates the great potential for high-level 5-ALA production *in vitro*. Limited by the relatively lower enzyme activities of cell extracts and complex engineered of recombinant strains, researchers were continually looking for more efficient and commercially viable approaches for 5-ALA synthesis. With the purification of the key enzymes in the metabolic pathway and elucidation of their structure and mechanism (Brown et al., 2018), it is possible to produce 5-ALA using a cell-free multi-enzyme catalysis system. Meng et al. (Meng et al., 2016) firstly constructed a cell-free multi-enzyme catalysis system based on the C4 pathway using the substrates succinic acid, glycine, and polyphosphate, catalyzed by the three enzymes, ALAS, succinyl-CoA synthase and polyphosphate kinase. Although the final titer of 5-ALA reached only 5.4 mM (0.71 g/L), the study demonstrated the possibility of cell-free multienzymatic synthesis. Subsequently, a cell-free semi-permeable process based on the C5 pathway was realized using glucose, sodium polyphosphate, ATP, NADPH, glutamate and nine enzymes (Zhao et al., 2019). Similar to previous research, the yield of 5-ALA was very low and the cost was very high, but this cell-free semi-permeable system firstly realized the recycling of enzymes and high-value cofactors for 5-ALA biosynthesis. In spite of the challenges faced by current studies, the considerable potential to realize commercial *in vitro* production by cell-free metabolic engineering cannot be ignored.

## 4 Detection of 5-ALA

Detection of 5-ALA is important for the study of synthetic pathways, metabolic mechanisms and applications of 5-ALA. In order to more easily measure the quantity of 5-ALA, detection methods based on Ehrlich’s reagent, fluorescent protein-based detection systems, liquid chromatography tandem-mass spectrometry (LC–MS/MS), high performance liquid chromatography (HPLC) and gas chromatography-mass spectrometry (GC–MS) have been developed.

### 4.1 Ehrlich’s Reagent Method

Ehrlich’s reagent was firstly used for the quantification of 5-ALA. It produces an adduct, that can be detected colorimetrically at 553 nm (Mauzerall and Granick, 1956; Tomokuni and Ogata, 1972). This method involves cell collection, extraction of intracellular components, and the Ehrlich reaction, which requires about 30 min for each sample (Mauzerall and Granick, 1956; Tomokuni and Ogata, 1972). Ehrlich’s reagent, as a classic detection method, has been widely accepted and used to measure the 5-ALA content, but the method suffers from severe sample background interference, in addition to being time-consuming and laborious.

### 4.2 Fluorescence Spectroscopy

5-ALA is stable only under acidic conditions. Currently, it is mostly sold in the form of hydrochloride and there is need for a simpler approach. The conversion of chemicals into easily detectable signals such as color or fluorescence has been a powerful tool for *de novo* metabolite monitoring and *in vitro* small molecule detection. Chung et al. (2005) proposed a combination of *hemA* with a green fluorescent protein gene (*egfp*) to realize the characterization and investigation of extracellular 5-ALA. Although the concentration of 5-ALA is reflected in the fluorescence intensity, the precise concentration of 5-ALA still depends on Ehrlich’s reagent. Recently, a fluorescence-based whole-cell system was developed for the simultaneous detection and production of 5-ALA in recombinant *E. coli* (Tan S. I. et al., 2019). In this method, a constitutive 5-ALA production strain with *R. sphaeroides* HemA (RshemA) and a fusion of super-folder-green fluorescent protein was established with an optimized promoter, medium, and fusion sites. The *de novo* quantification of intracellular and extracellular 5-ALA by monitoring the fluorescence of sfGFP fused to *RshemA* was also explored using dual promoters and dual plasmids (Tan S. I. et al., 2019). Additionally, to increase the understanding of the regulatory mechanism of 5-ALA in higher organisms, matrix isopotential synchronous fluorescence spectrometry and the first derivative technique were combined for the direct determination of δ-ALA in urine samples (Ajmal et al., 2019). The fluorescent derivatization of 5-ALA was based on the Hantzsch reaction. Matrix-isopotential synchronous fluorescence spectra were recorded along a matrix-isopotential trajectory, which combines the points of equal intensity in theoretical 3D contours. Maximum sensitivity and appropriate selectivity were ensured by the study of the experimental variables in the d-ALA bands, centered at its excitation and emission maxima (392.6 and 464.5 nm) (Ajmal et al., 2019). This method was comparable to conventional high performance liquid chromatography-fluorescence, with a detection limit of 5.4 ppb and lower linear-range limit of 18 ppb. These methods have improved effectiveness, enabling real-time detection with outstanding sensitivity, which is of great significance for ALA synthesis, detection and studies of the underlying metabolic mechanism.

### 4.3 Chromatography

Compared with a microbial fermentation broth with high 5-ALA content, samples with low 5-ALA concentrations such as blood, urine or tissue require more sensitive detection technology. The LC–MS/MS method was developed to simultaneously determine 5-ALA concentrations in fluids or tissues after solid-phase extraction, butanol derivatization, and quantification by selective reaction monitoring using ^13^C_5_, ^15^N-ALA and 2,4-^13^C_2_-PBG internal standards (Zhang et al., 2011; Dogan et al., 2019). The assay was highly sensitive for 5-ALA (LLOQ = 0.05-0.005 µM), and required ∼4 h from extraction to results. Later, a HPLC and GC–MS test method for 5-ALA detection was also developed (Ren et al., 2018; Hara et al., 2019) to obtain the real 5-ALA levels in microbial fermentation broth. Although these methods are sensitive and practical, more research is still needed to improve 5-ALA detection and decrease the assay costs.

## 5 Biological Effects and Applications of 5-Aminolevulinic Acid

### 5.1 Plant Growth Regulators, Herbicides and Insecticides in Agriculture

To date, 5-ALA has been demonstrated to act as a metabolic intermediate and also as a growth regulator in plant cultivation. It is regarded as a plant growth promoting hormone since many studies found that it could regulate the growth and development of higher plants. Instead of only focusing on the effects of 5-ALA on plant, more and more researchers began to use omics to explain its mechanism (Zheng et al., 2021). The role of 5-ALA in stimulating physiochemical processes in higher plants under stress, regulating plant growth and development in seed germination, vegetative growth and fruit coloring has been reviewed by Wu et al., in 2019 (Wu et al., 2018). Here, we mainly discussed the latest studies on its role in plant growth as well as the amelioration of the effects caused by various abiotic stresses.

5-ALA can selectively kill weeds without affecting monocotyledonous wheat and corn crops (Rebeiz et al., 1984). Therefore, a large amount of 5-ALA is used in plants or pests to exert the effects of herbicides and insecticides (Sasikala et al., 1994). For example, two different types of agricultural herbicides have been produced based on the principle that 5-ALA is necessary for plant life and cannot be synthesized in the dark. “Norfluron” can inhibit Glu-tRNA gene expression and prevent 5-ALA synthesis, resulting in a lack of 5-ALA leading to plant death (Kumar et al., 1999). Acifluorfen-methyl is a product that deregulates 5-ALA synthesis leading to a large accumulation of 5-ALA, thereby inhibiting the synthesis of heme and porphyrin compounds (Gullner and Dodge, 2000). However, higher concentrations required in herbicides and insecticides made it difficult to popularize the application or 5-ALA in this field based on current prices.

The abiotic stresses caused by environment, such as drought, salinity, high or low temperature, cause great losses to crop yields and social and economic aspects, and 5-ALA is helpful to alleviate these problems effectively. With the foliage application of 5-ALA at 75 mg/L, the activities of catalase, ascorbate peroxidase and superoxide dismutase of sunfower (*Helianthus annuus* L.) enhanced by 157.1, 90 and 80% under drought stress than no 5-ALA addition (Sher et al., 2021). It is reported that exogenous addition of a suitable concentration of 5-ALA can effectively alleviate weak light stress in tobacco seedlings (Li et al., 2019) and damages induced by UV-B in *Cajanus cajan* L. seedlings (Gupta and Prasad, 2021). Moreover, 5-ALA has shown a significant effect on alleviating abiotic stress caused by low temperature (Yan et al., 2020) and salinity (Islam et al., 2021; Wu et al., 2021) and so on in plants, which were summarized in detail in a recent review (Rhaman et al., 2021).

### 5.2 Photosensitizers and Cosmetics

Many studies have investigated fluorescence-guided resection of malignant tumors based on 5-ALA and protoporphyrin IX accumulation in tumors. This approach has become a well-established technique to facilitate greater extent of resection resulting in improved progression free survival (Stummer et al., 1998; Stummer et al., 2000; Pichlmeier et al., 2008; Stummer and Kamp, 2009; Rapp et al., 2012; Hadjipanayis et al., 2015; Guyotat et al., 2016). Additionally, 5-ALA was used with varying success in tumors such as meningiomas, medulloblastomas, ependymomas, and metastatic carcinoma (Millesi et al., 2016;Zhang C. et al., 2019; Wainwright et al., 2019). Other applications in medicine are being constantly developed, in addition to the applications related to cancer. Recently, there have been some excellent reviews covering the research progress on 5-ALA-based treatment of tumors (Wainwright et al., 2019; Zhang C. et al., 2019), and will not be repeated here.

Photodynamic therapy (PDT) with 5-ALA has been demonstrated as an effective treatment for severe acne in medical and cosmetic field (Itoh et al., 2000; Gold and Goldman, 2004), and recent studies have further researched the concentration of 5-ALA administration in different cases (Serini et al., 2019; Zhang et al., 2020b). Additionally, 5-ALA or its derivatives and ferric citrate compounds also are the main ingredient of disease prevention and improvement agent. A recent research demonstrated that 5-ALA combined with sodium ferric citrate could effectively improve aerobic capacity and voluntary exercise training achievement in older women over 75 years (Masuki et al., 2016; Ichihara et al., 2021). 5-ALA was also developed as a combined therapeutic-diagnostic agent in dentistry based on the strong dual modality of 5-ALA as lethal to cariogenic bacteria through photodynamic inactivation and enhancing LIF intensity for identification of dental caries (Lashkari et al., 2019). However, 5-ALA has a complex mechanism of action in the body, and the metabolic mechanism of 5-ALA conversion is still unclear. These unresolved issues led to a more prudent acceptance process of related products containing 5-ALA.

### 5.3 Promising Animal Feed Additives

It was found that adding appropriate amounts of 5-ALA to animal feed can significantly improve their growth. In invertebrate aquaculture, dietary administration of 5-ALA effectively improved the immune system, ATP levels and acute hepatopancreatic necrosis disease resistance in pacific white shrimp (Direkbusarakom et al., 2021). Dankook University studied the effects of 5-ALA on the growth performance, blood status, and immune system of weaned piglets, sows, broilers and hens in South Korea. These results showed that adding appropriate amounts of 5-ALA to the diet can increase the hemoglobin concentration and iron in the serum, the levels of lymphocyte subsets, the quality of immune organs and the immune performance of these animals (Chen Y. J. et al., 2008; Chen Y. et al., 2008; Wang et al., 2009). Similarly, 5-ALA had the same effect on cows (Hendawy et al., 2019). Our own recent research work tried to efficiently enrich feed with 5-ALA by developing a solid-state fermentation process based on *S. cerevisiae* (Mao et al., 2020). In this work, 5-ALA not only plays a role in improving the animal’s growth performance and immune system, but yeast cells can be used as feed protein to provide nutrition for animals. In summary, it was concluded that 5-ALA could indeed be used as an animal feed additive to improve the iron status and immune response in livestock, which was also reviewed recently by Hendawy et al. recently (Hendawy et al., 2020).

## 6 Discussion and Outlook

### 6.1 Exploration of Production Hosts With Natural Advantages

According to the Comprehensive Enzyme Information System of BRENDA, there are 147 known organisms with 5-aminolevulinate synthesis ability, and we can assume that many natural hosts have not yet been developed to synthesize 5-ALA. In the future, high-yield production of 5-ALA can be achieved by fully screening natural hosts with innate advantages in 5-ALA synthesis. Furthermore, efficient 5-ALA synthesis can also be achieved through further metabolic engineering to transform the current model strains to give full play to their natural advantages. For example, *C. glutamicum* can naturally accumulate amino acids and lacks a glycine cleavage pathway, making it a highly attractive potential biosynthetic host (Jorgensen et al., 2012). It was shown that the activation of native antioxidant defense systems alleviated the deregulation of ROS metabolism due to 5-ALA accumulation and further improved 5-ALA tolerance and synthesis (Zhu et al., 2019). Therefore, hosts such as *C. glutamicum* and *S. cerevisiae* with the advantages of natural resistance to high concentrations of organic acids can be further explored as chassis cells for 5-ALA synthesis. Fully exploiting and utilizing the natural advantages of hosts to build a 5-ALA artificial cell factory may be an effective way to synthesize 5-ALA.

### 6.2 In-Depth Analysis and Understanding of Relevant Mechanisms of 5-ALA Biosynthesis

Although the synthesis and application of 5-ALA has been extensively studied, there are still some challenges that need to be resolved, such as the regulation and transport mechanisms in 5-ALA biosynthesis and the metabolic mechanisms related to 5-ALA application. First, the feedback regulation of ALAS or GluTR by heme exists in plants, animals and microorganisms, and influences the 5-ALA biosynthesis and host cell growth. In addition, feed-back regulation by downstream products in plant cells regulates the biosynthesis of 5-ALA in an opposite manner. However, the mechanism of feedback regulation by these factors and heme has not been elucidated. Secondly, the cellular transport capacity is also crucial for 5-ALA biosynthesis, which has been proved in previous research (Feng et al., 2016). However, Transport mechanisms of 5-ALA are not fully clear and need to be studied further. Detailed knowledge of the metabolic mechanisms of 5-ALA will resolve the safety issues and improve its industrial applications.

### 6.3 Improving 5-Aminolevulinic Acid Synthesis Using Artificial Microbial Consortia

Recently, a growing fraction of research has transitioned towards employing a modular co-culture engineering strategy using multiple microbes growing together to facilitate a “divide-and-conquer” approach for chemical biosynthesis. Artificial microbial consortia have been constructed and applied to produce bio-based chemicals (Lu et al., 2019), natural products (Wang R. F. et al., 2020) and bioenergy (Jiang et al., 2019). According to the latest research progress on metabolic engineering for the synthesis of 5-ALA (Supplementary Table S1), the high-level production 5-ALA through the C4 pathway requires the exogenous addition of glycine in all cases. Moreover, our research group confirmed the effect of the addition of different glycine concentrations on the synthesis of 5-ALA in *C. glutamicum* (Zou et al., 2017), and the supply of glycine is the key limiting factor for 5-ALA synthesis in the C4 pathway (Ren et al., 2018). However, the addition of glycine not only increases the cost of the product, but the toxic effects of high glycine concentrations on the cells will also inhibit the growth of engineered bacteria and reduce the production efficiency. Moreover, 5-ALA is synthesized from the precursors glycine and succinyl-CoA in the C4 pathway, whose balanced supply is the key to the efficient synthesis of 5-ALA. To achieve precise control of the two precursors and the efficient synthesis of 5-ALA, synthetic microbial consortia can be constructed by placing the synthesis pathway modules of glycine and succinyl-CoA into different engineered strains. Based on the flexible optimization of the metabolic module within a single strain, combined with the adjustment of the inoculation volume and fermentation process of the microbial consortia, the artificial microbial consortia may be used as a potential method to synthesize 5-ALA. This approach can save costs and reduce the burden imposed on the cellular metabolism, solve the problem of flux optimization of nonlinear synthetic pathways, and achieve precise control of synthetic pathways.

## Supplementary Material

Supplementary Table S1 Recent researches on the biosynthesis of 5-aminolevulinic acid *in vivo* or *in vitro*.

## Abbreviations

5-ALA, 5-aminolevulinic acid; ALAS, 5-aminolevulinic acid synthase; pyridoxal PLP, 5′-phosphate; GluTS, glutamyl–tRNA synthetase; GluTR, glutamyl-tRNA reductase; GSA, L-glutamic acid 1-semialdehyde; GSA-AM, glutamate-1-semialdehyde aminotransferase; ALAD, 5-aminolevulinate dehydratase; OdhI, oxoglutarate dehydrogenase inhibitor; GS, glutamate dehydrogenase; asRNAs, antisense RNA; PMP, pyridoxamine 5′-phosphate; PEP, phosphoenolpyruvate; ROS, reactive oxygen species; LA, levulinic acid; 5′-UTRs; 5′-untranslated regions; CRISPRi, CRISPR interference.
