# Supplementary material for: Natural 5-Aminolevulinic Acid: Sources, Biosynthesis, Detection and Applications
Source: Front Bioeng Biotechnol. 2022 Feb 25;10:841443. doi: 10.3389/fbioe.2022.841443 (PMC8913508; doi:10.3389/fbioe.2022.841443)
Supplement: Supplementary file 1 [file Table1.docx]

**Supplementary Table S1 Recent researches on the biosynthesis of 5-aminolevulinic acid *in vivo* or in *vitro*.**

|  | **Original Strain** | **Strains and Strategies** | **Pathway** | **Main Substrates** | **5-ALA Titer (g/L)** | **Productivity (g/L/h)** | **Reference** | |
| --- | --- | --- | --- | --- | --- | --- | --- | --- |
| ***In Vivo*** | | | | | | | | |
| *E.coil* | *E. coli BL21*(DE3) | Co-expression of chaperone (GroELS) with hemA from *R*. *capsulatus*, supplement of ferric ion andoptimized glucose-glycerol mixed carbon source | C4 | Glucose, glycerol, glycine, succinate and ferric ion | 15.6 ^a^ | 0.557 ^a^ | (Yu et al., 2022) | |
|  | *E. coli* BW25113 | Expression of *hemA*, downregulation of *hemB* expression with CRISPRi, deletion of *sdhA* and *iclR*, microaerobic and aerobic conditions | C4 | Glycerol | 6.93^a^ | — | (Miscevic et al., 2021) | |
|  | *E. coli* BL21(DE3) | Ingratiation PdxY to enhance the production of PLP, expression of *hemA* from *R*. *capsulatus* | C4 | Glucose, glycine, pyridoxal | 8.21^a^ | 0.228^b^ | (Xue et al., 2021) | |
|  | *E. coli* BL21(DE3) | Downregulation of *hemB* expression by antisense RNAs (asRNAs) | C4 | Glucose, succinic acid, glycine | 1.23 | 0.062 | (Ge F. L. et al., 2021) | |
|  | *E. coli* BW25113 | Co-overexpression of *katE*, *sodB* to degrade ROS | C4 | Glucose, succinic acid, glycine | 11.5^a^ | 0.523^b^ | (Zhu et al., 2019) | |
|  | *E. coli* BL21(DE3) | Expression of codon-optimized *hemA* with the T7 system, improvement of soluble form expression with TrxA and GroELS | C4 | Succinic acid, glycine | 5.66 | — | (Yu et al., 2019) | |
|  | *E. coli* BL21(DE3) | Construction of a quantified and regulated ALA production method with *sfGFP*, *hemA_R.S_* and *rhtA* | C4 | Glucose, succinic acid, glycine | 2.46 | 0.103 | (Tan et al., 2019) | |
|  | *E. coli* BL21(DE3) | Downregulation of *hemB* expression with glycine riboswitch at an unnatural pathway | C4 | Glucose, glycine | 0.242 | 0.012 | (Zhou et al., 2019) | |
|  | *E. coli* BL21(DE3) | Overexpression of *hemA*, construction of unnatural glycine pathway with *aceA* and *agxt* | C4 | Glucose | 0.521^a^ | 0.029^b^ | (Ren et al., 2018) | |
|  | *E. coli* Rosetta(DE3) | Co-overexpression of *hemA* and *serA*, improvement CoA pathway by *coaA^M^*, *coaD* and *dfp*, downregulation *hemB*, inaction of *sucCD* | C4 | Glucose | 2.81^a^ | 0.088^b^ | (Ding et al., 2017) | |
|  | *E. coli* Rosetta(DE3) | Overexpression of *hemA* and taking short-term DO shock during aerobic fermentation | C4 | Glucose, succinic acid, glycine | 9.4^a^ | 0.427^b^ | (Yang et al., 2013) | |
|  | *E. coli* MG1655 | Overexpression of *hemA* and *hemO* from *R. palustris* respectively | C4 | Glucose, succinic acid, glycine | 6.3^a^ | 0.263^b^ | (Zhang et al., 2013) | |
|  | *E. coli* Rosetta(DE3) | Overexpression of *hemA* from *A. radiobacter* and optimization of fermentation conditions | C4 | Glucose, succinic acid, glycine | 6.5^a^ | 0.295^b^ | (Fu et al., 2010) | |
|  | *E. coli* BL21(DE3) | Addition of inhibitor D-glucose | C4 | Succinic acid, glycine, LA, D-glucose | 3.1^a^ | 0.107^b^ | (Liu et al., 2010) | |
|  | *E. coli* Rosetta (DE3) | Overexpression of *hemA* with a two-stage pH controlling fed-batch culture system | C4 | Glucose, succinic acid, glycine | 6.6^a^ | 0.236^b^ | (Fu et al., 2008) | |
|  | *E. coli* Rosetta (DE3) | Overexpression of *hemA* and optimization of fermentation conditions, inhibitors | C4 | Glucose, succinic CoA, glycine, xylose | 7.3^a^ | 0.243^b^ | (Lin et al., 2009) | |
|  | *E. coli* BL21 | Screen of optimum medium and optimization of precursors, pH | C4 | Glucose, succinic acid, glycine | 5.15^a^ | — | (Choi et al., 2008) | |
|  | *E. coli* Rosetta (DE3) | Optimization of fermentation conditions, precursors and inhibitors | C4 | Glucose, succinic acid, glycine | 3.8^a^ | 0.211^b^ | (Fu et al., 2007) | |
|  | *E. coli* BL21(DE3) | Optimization of fermentation conditions, precursors and inhibitors | C4 | Glucose, succinic acid, glycine, LA | 1.3^a^ | 0.046^b^ | (Chung et al., 2005) | |
|  | *E. coli* MG165 | Optimizing the expression of *hemA* at two strains with the concentration of initial substances and induction | C4 | Succinic acid, glycine, glucose | 5.2^a^ | 0.433^b^ | (Xie et al., 2003) | |
|  | *E. coli* BL21(DE3) | Overexpression of *hemA* from *Bradyrhizobium japonicum* and optimization of fermentation conditions | C4 | LA, succinic acid, glycine | 3.2^a^ | 0.229^b^ | (Choi et al., 1999) | |
|  | *E. coli* MG1655 | Using CICHE integrate *hemA* and *hemL* into genome with 98 copy-number to achieve plasmid-free | C5 | Glucose | 4.55 | 0.063 | (Cui et al., 2019) | |
|  | *E. coli* BL21(DE3) | Co-overexpression of *HemA1*, *pgr7* from *Arabidopsis thaliana* | C5 | Glucose, glutamate | 7.642 | 0.509 | (Zhao and Zhai, 2019) | |
|  | *E. coli* PfliCT7RE | Co-overexpression of *hemA*, *hemL* *hemD hemF* and *rhtA*, downregulation of *hemB* expression with fliCp, strength of the cofactor PLP with *pdxH*, deletion of *recA* and *endA* to improve plasmid stability, a two-stage pH fermentation strategy | C5 | Glucose | 5.25^a^ | 0.159^b^ | (Zhang et al., 2019) | |
|  | *E. coli* DH5α | Co-overexpression of *hemA*, *hemL* and the PHBV synthetic pathway to produce ALA and PHAs | C5 | Glucose | 3.0 | — | (Zhang X. et al., 2018) | |
|  | *E. coli* W | Co-overexpression of *hemA^mu^*^t^, *hemL*, *gltA* and *aceA*, inaction of *sucA* | C5 | Glucose | 3.4 | 0.190 | (Noh et al., 2017) | |
|  | *E. coli* BL21 (DE3) | Co-overexpression of *hemA*, *hemL*, *hemF*, and *hemD*, analysis of the mechanism of RyhB | C5 | Glucose | 4.05^a^ | 0.127^b^ | (Zhang et al., 2016) | |
|  | *E. coli* BL21 (DE3) | Co-overexpression of *hemA*, *hemL*, *hemD* and *hemF*, inaction of *hemB* | C5 | Glucose | 3.25^a^ | 0.102^b^ | (Zhang et al., 2015) | |
|  | *E. coli* DH5α | Co-overexpression of *hemA*, *hemL* and *rhtA* | C5 | Glucose | 4.13 | 0.086 | (Kang et al., 2011) | |
| *C. glutamicum* | *C.glutamicum* F343 | Deletion of *gdhA* and *aceA*, overexpression hemA from *R. capsulatus* | C4 | Glucose and glycine | 5.6 | 0.133 | (Ge F. et al., 2021) | |
|  | *C. glutamicum* ATCC 13032 | Screen of *hemA* from different source and changing RBS, overexpression *ppc* with moderate RBS, usage of hydrolyzed cassava bagasse, corn starch hydrolysate and beet molasses replacing glucose at fed-batch fermentation | C4 | Hydrolyzed cassava bagasse, glycine | 18.5^a^ | 0.385^b^ | (Chen et al., 2020) | |
|  | *C. glutamicum* ATCC 13032 | Co-overexpression of *hemA*, *serB*, serC, *serA^D197^* and *glyA* | C4 | Glucose, glycine | 3.4 | — | (Zou et al., 2017) | |
|  | *C. glutamicum* ATCC 13032 | Co-overexpression of *hemA* and *rhtA,* deletion of *ldhA*, *pqo*, *cat*, *pta*, *ackA*, *pck* and *pbp1b* | C4 | Glucose, glycine | 7.53^a^ | 0.251^b^ | (Feng et al., 2016) | |
|  | *C. glutamicum* ATCC 13032 | Overexpression of *hemA* and *rhtA*, deletion of *sucCD* | C4 | Glucose, glycine | 14.7^a^ | 0.920^b^ | (Yang et al., 2016) | |
|  | *C. glutamicum* ATCC 13032 | Modulation of the flux of ATP, NADPH and PLP, control the expression of *odhA* with auto-inducible metabolic engineering, moderate expression of *rhtA* | C5 | Glucose | 3.16 | 0.049 | (Zhang et al., 2020) | |
|  | *C. glutamicum* ATCC 13032 | Co-overexpression of *hemA^M^*, *hemL*, *rhtA* and *odhI^T14A/T15A^*，which inactivates ODHC to improvethe flux redistribution of TCA | C5 | Glucose | 2.9 | 0.060 | (Ko et al., 2019) | |
|  | *C. glutamicum* S9114 | Co-overexpression of *hemA*, *hemL* and *rthA*, inaction of *ncgl1221*, *lysE*, and *putP* | C5 | Glucose | 0.895 | 0.012 | (Zhang and Ye, 2018) | |
|  | *C. glutamicum* ATCC 13032 | Co-overexpression of *hemL* and *hemA*, regulation of DO and the concentration of Fe^2+^ | C5 | Glucose | 1.79 | 0.012 | (Yu et al., 2015) | |
|  | *C. glutamicum* ATCC 13032 | Co-overexpression of *hemL* and *hemA*, perturbation of glutamate and heme by adding PG and DP | C5 | Glucose | 2.2 | 0.046 | (Ramzi et al., 2015) | |
| *S. cerevisiae* | *S. cerevisiae* YPH499 | Optimization of glycine and LA, overexpression of ACO2 and HemA1, develop measure method by GC-MS | C4 | LA, glucose, glycine | 0.00136 | — | (Hara et al., 2019) | |
|  | *S. cerevisiae* INV_SC_l | Co-expression the C4 pathway and the C5 pathway | C4, C5 | Glucose, succinic acid, glycine | 0.526 | — | (Zhang J. L. et al., 2018) | |
| *Others* | *Yarrowia lipolytica* | Co-expression the C4 pathway and the C5 pathway, Optimization of fermentation conditions | C4, C5 | Glucose, glycerol, LA, glycine and glutamate | 2.217 ^a^ | 0.025^b^ | (Cui et al., 2021) | |
|  | *Shewanella*  *oneidensis* MR-1 | Downregulation of *hemB* expression in the C5 pathway, integration of *hemA* and *gro*ELS to the chromosome in the C4 pathway | C4, C5 | Glucose, succinic acid, glycine | 0.207 | 0.009 | (Yi and Ng, 2021) | |
|  | *Bacillus subtilis* 1A747 | Co-expression of *hemA* and *hemL* | C5 | — | 0.069 | 0.001 | (Liu et al., 2020) | |
|  | *Streptomyces coelicolor* A3(2) | Erexpression of *hemA* from *R. sphaeroides*, optimization of medium composition at *Streptomyces coelicolor* | C4 | Glucose, succinic acid, glycine | 0.137^a^ | 0.005^b^ | (Tran et al., 2019) | |
|  | *Propionibacterium*  *acidipropionici* | Optimization of fermentation conditions, precursors | C4 | Glucose, succinic acid, glycine | 7.7 | 0.053 | (Sonhom et al., 2012) | |
|  | *P. acidipropionici* TISTR442 | Addition of LA and glycine at *Propionibacterium acidipropionici* | C4 | Glucose, glycine | 0.405 | — | (Kiatpapan et al., 2011) | |
|  | *Propionibacterium freudenreichii* | Overexpression of *hemA* from *R. sphaeroides* and the proportion of glycine and LA | C4 | Glucose, glycine, LA | 1.04 | — | (Kiatpapan and Murooka, 2001) | |
|  | *Rhodopseudomonas*  *palustris* KG31 | Optimization of LA, glycine and succinate at *R. palustris* | C4 | Glucose, succinic acid, glycine, LA | 0.024 | — | (Saikeur et al., 2009) | |
|  | *Rhodobacter sphaeroides* CR 606 | *R. sphaeroides* mutants | C4 | Glucose, glycine | 3.60 | — | (Kamiyama et al., 2000) | |
|  | *R. sphaeroides* ATCC17023 | Optimizing the proportion of glucose, succinic acid and glycine | C4 | Glucose, succinic acid, glycine | 3.55 | — | (Liu et al., 2016) | |
|  | *Chlorella. regularis* YA-603 | *C. regularis* mutants and addition of glycine | C4 | Glucose, glycine, LA | 0.51 | 0.011 | (Ano et al., 1999) | |
|  | *Rubrivivax benzoatilyticus* PS-5 | Optimizing the proportion of substrates acetic acid, propionic acid and butyric acid and pH-control strategy | C4 | Succinic acid, glycine, LA | 0.038 | — | (Sattayasamitsathit and Prasertsan, 2014) | |
| ***In vitro*** | | | | | | | |  |
|  |  | Semipermeable reaction system with recycled multi-enzymes *in vitro* | C5 | Glucose, sodium polyphosphate,  ATP, tRNA, glutamate, NADPH | — | — | (Zhao et al., 2019) | |
|  |  | Cell free multi-enzyme catalysis *in vitro* | C4 | Succinic acid, glycine, PLP | 0.708 | — | (Meng et al., 2016) | |

“a” and “b” represent the 5-ALA titer or productivity obtained by fermentation in a bioreactor. “-” represents information that was not mentioned in the ref
